# High flow nasal oxygen therapy for COVID 19: an unusual complication

**DOI:** 10.1186/s42077-022-00242-1

**Published:** 2022-05-04

**Authors:** Tushar Kumar, Amit Tirkey, P. K. Bhattacharya, U. Suwalka, Ladhu Lakra

**Affiliations:** 1 Department of Anaesthesiology – Trauma, Rajendra Institute of Medical Sciences, Ranchi, Jharkhand 834009 India; 2 Department of Anaesthesiology, Rajendra Institute of Medical Sciences, Ranchi, Jharkhand 834009 India; 3 Department of Critical Care, Rajendra Institute of Medical Sciences, Ranchi, Jharkhand 834009 India

**Keywords:** COVID-19, HFNO, Hypoxia, Ulcers

## Abstract

**Background:**

Acute hypoxemic respiratory failure is the most common complication of COVID 19 infection. Newer ways for oxygen therapy were explored during this pandemic. High flow nasal oxygenation (HFNO) emerged as a novel technique for oxygenation and prevented the need for invasive mechanical ventilation during hypoxia among COVID patients. Using high flow oxygen dries the nasal mucosa and leads to skin disruption. We are presenting this case as this complication has not been reported anywhere to our knowledge.

**Case presentation:**

Here we present a case of a 62-year-old male, who was on HFNO for a long time as a part of treatment for COVID 19 and developed ulceration in the nasal septa. Patient belonged to a geriatric age group and had diabetes mellitus. Close monitoring by ICU (intensive care unit) staff was a big problem during this pandemic. Daily physical assessment, good nutrition, and daily dressing with plastic surgery consultation helped treat our patient.

**Conclusions:**

Geriatric patients with other co-morbidities are vulnerable to mucosal injury. Even in COVID era, everyday general physical surveillance is very vital in such patients to prevent these complications. During this pandemic close monitoring of patients suffered due to scarcity of ICU staff. In spite of that, it is a must to ensure daily physical surveillance and good supplemental nutrition especially in geriatric patients.

## Background

In the wake of the COVID 19 pandemic, unique ways of oxygen therapy techniques were searched. Acute hypoxemic respiratory failure is the most common complication of COVID 19 infection. High flow nasal oxygenation (HFNO) emerged as a novel technique for oxygenation and prevented the need for invasive mechanical ventilation (IMV) in hypoxia among COVID patients. Though there is paucity of evidence to support the effectiveness of HFNO therapy, but it has been used worldwide enthusiastically. Here we present a case of a 62-year-old male, who was on HFNO for a long time as a part of treatment for COVID 19 and developed ulceration in the nasal septa. The case report adheres to the CARE guidelines.

## Case presentation

A 62-year-old male patient was brought to the emergency room with a 2-day history of fever and dyspnea on exertion. His past medical history included type 2 diabetes and hypertension. The patient was non-alcoholic and non-smoker and had previously been taking metformin and amlodipine. His vital signs included non-invasive blood pressure of 150/88 mmHg, pulse rate of 92 beats/min, respiratory rate of 20/min, oxygen saturation of 90% on room air, and a temperature of 99 °F. Relevant findings on physical examination included regular, rapid heart rhythm during auscultation. Bilateral breath sounds with rhonchi were heard. There was no lower-limb swelling or calf tenderness. An electrocardiogram taken in the emergency room revealed sinus tachycardia at a rate of 112 bpm with no acute ST-T segment changes. A chest x-ray was normal although the patient was feverish, tachypnic, bilateral chest rhonchi, PaO2 = 90%, and PCR was positive. Laboratory data reported white blood cell count of 12,500/cu mm, hemoglobin of 14.2 g/dL, and platelet count of 1.58 × 10^5^/cu mm. The basic metabolic panel reports were within normal range except for the blood sugar of 260 mg/dL. D-dimer was elevated at 1800 ng/mL. After a throat swab for SARS-CoV-2, RT-PCR test was sent from the emergency room. Patient was shifted to COVID-suspected Ward after primary care. His COVID 19 RT-PCR report came out to be positive the next day. He was then shifted to COVID ICU for further management.

**Fig. 1:**
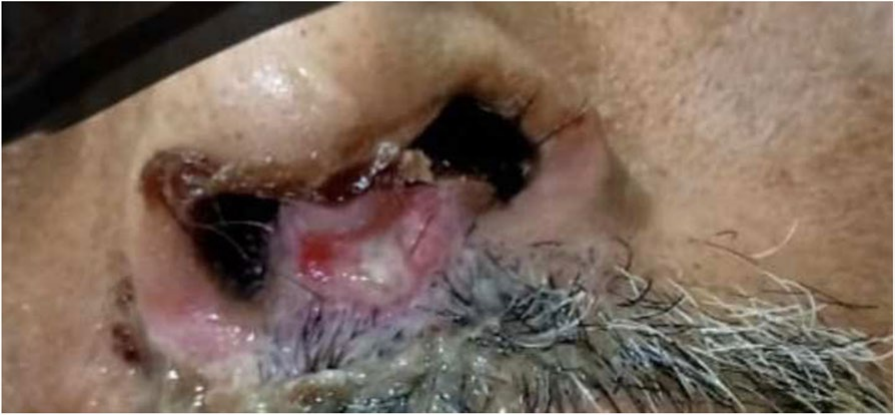
Mucosal breech in nasal septa

In the ICU, patient was managed with Venturi mask with FiO2 50%, nebulization with bronchodilators, remdesivir, antibiotics, steroid, vitamins C, zinc tablets, low molecular weight heparin ( LMWH), enteral nutrition, and other supportive care. Patient remained febrile and hypoxemia further worsened. HFNO was initiated on day 3 of ICU admission with FiO2 100% at 60L/ min flow at 36 °C temperature and humidification was done with sterile water. His PaO2 was 86 mmHg and did not show further deterioration. His blood sugar began to rise and his average reading was 324mg/dL. He was managed with injection, regular and long-acting insulin. He turned COVID negative 15 days after admission in our ICU. He continued to have the requirement for high flow oxygen. He had regular chest physiotherapy with antibiotics and supportive care. He was shifted to a non-COVID ICU for further management. He continued to remain hypoxemic without HFNO support. He was managed with intermittent proning along with HFNO and other medications for 22 days. In due course, he developed sloughing of the nasal septa and ear pinna (shown in Fig. 1). The sloughing of the nasal septa started with skin aberration and gradually extended to the mucosa. It looked like a pressure ulcer due to tight-fitting nasal prong. He was shifted to the ward with a face mask and fresh gas flow of 2 L/ min. Gradually with proper dressing and treatment, healing started and later patient was discharged on oral hypoglycemics and bronchodilators.

## Discussion

Acute hypoxemic respiratory failure is the most common complication of COVID 19 infection (Xia et al. 2020). High flow nasal oxygenation (HFNO) emerged as a novel technique for oxygenation and prevented the need for invasive mechanical ventilation (IMV) in hypoxia among COVID patients (Calligaro et al. 2020). Respiratory support is applied to maintain proper oxygenation and promote alveolar ventilation in hypoxemic respiratory failure when supplemental oxygen is failed as first-line treatment (Nishimura 2016). The anatomy of our oropharyngeal space is such that inspired air is humidified and warmed with each breath. Dry or poorly humidified oxygen may arise some unwanted concerns such as dry nose, dry throat, and nasal pain, and consequent intolerance to oxygen therapy. Unconditioned medical gas increases airway resistance in order to protect the lungs from dry or cold-inspired air by reducing air flow in the upper airways and trachea (Chanques et al. 2009). Inspired of an open system, HFNO is able to generate positive end-expiratory pressure (PEEP). Hedge and Prodhan reported cases of pneumothorax and pneumomediastinum in patients receiving HFNO therapy (Hegde and Prodhan 2013). Breach in mucosa and sloughing of skin is rarely seen with HFNO. Our patient received HFNO for a very long time. Collagen fibers and elastin cells are reduced in number and loses its strength in old age. They are easily damaged and healing is delayed (Payne 2020). Nasal prongs in our case are made up of silicone and straps are made of a softer, tubular material with Stretchwise technology. There is paucity of evidence against these nasal prongs and straps to cause such harm to the patient. Rathore et al. reported a case of pressure ulcer over nasal bridge because of face mask while using non-invasive ventilation (NIV). The facial mask was applied continuously for NIV support, without intermittent loosening of straps or close monitoring of the underlying skin (Rathore et al. 2016). Breakdown of skin in nares and pressure ulcers over the septa of the nose and behind ear pinna due to nasal prongs and straps, respectively, have not been reported yet. The aim of this case report is to highlight the importance of early identification and prevention of skin aberration associated with HFNO. A disease which became a social stigma for some time led to fear and ignorance of critical COVID patients. Due to segregation and isolation in COVID ICU, it was difficult to inspect the minute details in the PPE (personal protective equipment) kit. These kinds of ulcerations were not anticipated with the use of HFNO cannula. It is very important that nursing staff maintain sufficient documentation regarding time intervals for HFNO usage on all patients in their care and promptly communicate any unwanted event to other nurses and doctors. Also, timely use of proper antibiotics, proper dressing, and glucose control promoted proper healing.

## Conclusions

During this COVID pandemic, HFNO emerged as a promising technique for oxygen therapy. The use of HFNO, when unmonitored, can lead to several complications, including pneumothorax, pneumomediastinum, poor ventilators outcomes, and mucosal ulcers. Documentation, communication, and early identification by careful surveillance of ulcers are the key to preventing this complication.

In a limited resource facility, it is prudent to anticipate the unexpected. We could have escaped such complications by improving communication along with proper training and education of all healthcare professionals. Even in the time of acute shortage of staff and high patient load, we should not forget the very basic pillars of critical care management.

## Data Availability

Data sharing is not applicable to this article as no datasets were generated or analyzed during the current study; however, patient case record file is present in the hospital medical record department.

## Abbreviations

HFNO: High flow nasal oxygen
IMV: Invasive mechanical ventilation
NIV: Non-invasive ventilation
LMWH: Low molecular weight heparin
PEEP: Positive end-exploratory pressure.
PPE: Personal protective equipment
